# Medication Use Among Pregnant Women With SARS-CoV-2 Infection and Risk of Hospitalization—A Study in Two Brazilian Hospitals

**DOI:** 10.1155/2024/8915166

**Published:** 2024-06-06

**Authors:** Ricardo Rohweder, Natálya G. Pereira, Bruna H. Micheletti, Jéssica Mosello, Júlia R. M. Campos, Matheus G. Pereira, Cristina N. Santos, Natália L. Simões, Regina L. B. Matielo, Lisandra S. Bernardes, Maria L. R. Oppermann, Maria C. O. Wender, Angela Lupattelli, Hedvig Nordeng, Lavinia Schuler-Faccini

**Affiliations:** ^1^ Graduate Program in Genetics and Molecular Biology Department of Genetics Universidade Federal do Rio Grande do Sul, Porto Alegre, Brazil; ^2^ Department of Obstetrics and Neonatology Hospital e Maternidade SEPACO, São Paulo, Brazil; ^3^ Center for Klinisk Forskning and Afdeling for Kvindesygdomme, Graviditet og Fødsel North Denmark Regional Hospital, Hjørring, Denmark; ^4^ Gynecology and Obstetrics Service Hospital de Clínicas de Porto Alegre, Porto Alegre, Brazil; ^5^ PharmacoEpidemiology and Drug Safety Research Group Department of Pharmacy University of Oslo, Oslo, Norway

## Abstract

There is limited evidence about the use of medications among pregnant women with COVID-19, as well as risk factors for hospitalization due to COVID-19 in pregnancy. We aimed to describe the use of medications among SARS-CoV-2-positive pregnant women at the time around infection and identify predictors for hospitalization due to COVID-19 in two hospitals in Brazil. This is a hospital record-based study among pregnant women with positive SARS-CoV-2 tests between March 2020 and August 2022 from two Brazilian hospitals. Characteristics of sociodemographic, obstetrical, and COVID-19 symptoms were extracted retrospectively. The prevalence use of medications was based on self-reported use, and this was administered at the hospital. Logistic regression was used to estimate predictors of hospitalization due to COVID-19. There were 278 pregnant women included in the study, of which 41 (14.7%) required hospitalization due to COVID-19. The remaining 237 (85.3%) had mild symptoms or were asymptomatic. Most of the women had the infection in the third trimester (*n* = 149; 53.6%). The most prevalent medications used across all trimesters were analgesics (2.4% to 20.0%), antibacterials (15.0% to 23.1%), and corticosteroids (7.2% to 10.4%). Pre- or gestational hypertensive disorder (odds ratio (OR) 4.94, 95% confidence interval (CI) 1.65, 14.87) and having at least one dose of vaccine against SARS-CoV-2 (OR 0.13, 95% CI 0.04, 0.39) were associated with hospitalization due to COVID-19. Analgesics, antibacterials, and corticosteroids were the most frequently used medications among pregnant women with COVID-19. Women with hypertensive disorders have almost a five-fold increased risk of hospitalization due to COVID-19. Vaccination was the strongest protective factor for severe COVID-19. The COVID-19 vaccination among pregnant women should be promoted, and pregnant women diagnosed with COVID-19 who have hypertensive disorders should be closely monitored.

## 1. Introduction

With the emergence of the SARS-CoV-2 and the consequent COVID-19 pandemic, concerns about the impact of the infection among pregnant women have rapidly appeared. Studies have consistently shown that pregnant women have a higher risk of more severe COVID-19 than nonpregnant women of reproductive age [1]. Moreover, infection during pregnancy increases maternal morbidity and mortality [2]. SARS-CoV-2 infection during pregnancy has consistently been associated with an increased risk for premature birth, pre-eclampsia, stillbirth, and neonatal and maternal mortality [3]. Brazil has the second-highest number of reported deaths due to COVID-19, and by November 2021, more than 7400 pregnant women were hospitalized due to COVID-19 [4]. In 2020, the first year of the COVID-19 pandemic, there was a 40% increase in maternal mortality compared to the expected number based on the previous 5 years [5].

Understanding the risk factors associated with severe COVID-19 course and adverse outcomes is essential in guiding health policies, allocating resources and defining treatment criteria based on the best available evidence. For the general population, respiratory diseases, diabetes, cardiovascular conditions, hypertension, and obesity are well-recognized risk factors for severe COVID-19 [6–9]. Noteworthy, among pregnant women, some factors increase the risk for severe COVID-19: Allotey et al. [1] found that pregnant women ≥ 35 old have a 1.5-fold increased risk, body mass index ≥ 30 has a 1.8-fold increased risk, chronic hypertension has a 1.7-fold increased risk, and pre-existing diabetes and gestational diabetes have 2.9-fold and 1.6-fold increased risks, respectively. Even more notably, the authors found that pre-eclampsia has a 5.3-fold increased risk [1]. The magnitude of risk, however, may vary considerably by the infection rates between countries and the healthcare systems and mitigation strategies that these countries utilize.

Since the start of the pandemic, there has been a strong focus on medications to treat COVID-19. Studies have investigated repurposing medications to treat COVID-19 [10–12], but there is a lack of information about the use and safety of the use of these during pregnancy, and pregnant women have commonly been excluded from such clinical trials [13, 14]. Developing guidelines for the management of pregnant women diagnosed with COVID-19, and keeping them updated, has been a challenging, ongoing task [15, 16].

This work complements the knowledge about how medications are used by women with COVID-19 during pregnancy and risk factors for severe COVID-19 throughout pregnancy. In specific, our aim is two-fold: (i) to describe the prevalence of the use of medications around the time of COVID-19 diagnosis among pregnant women by severity and by trimester and (ii) to identify risk factors for hospitalization due to COVID-19 among women in two Brazilian hospitals.

## 2. Materials and Methods

### 2.1. Design and Settings

This is a retrospective cohort study, performed with data from two tertiary-care Brazilian hospitals, Hospital de Clínicas de Porto Alegre (HCPA), located in Porto Alegre, and SEPACO Hospital e Maternidade, located in São Paulo. The HCPA is a general public university hospital that offers care through the national public health system (Sistema Único de Saúde (SUS)), with approximately 2800 births per year. SEPACO is a philanthropic hospital, focused on high-complexity care, through health insurance plans and private costs. The maternity hospital offers prenatal services, including foetal surgery, with approximately 4400 births per year.

### 2.2. Study Period

The enrollment period started on March 8, 2020, and ended on August 31, 2022. In this period, some changes regarding the surveillance and prevention of COVID-19 in pregnancy have taken place and are worth noting. Considering the greater risk that pregnant women have for adverse outcomes when infected with SARS-CoV-2, since August 31, 2020, the national guidelines for the care of pregnant women recommended testing those who had symptoms of COVID-19, as well as asymptomatic pregnant women who require admission to the hospital, regardless of the reason for hospitalization. Therefore, from that date onwards, there was intense surveillance of infections among pregnant women. Moreover, as of March 15, 2021, vaccination for COVID-19 was made available to pregnant women who had comorbidities, and as of April 26, 2021, to all pregnant women.

### 2.3. Ethics

This research was submitted to Plataforma Brasil and approved by the Ethics Committee under the Certificate of Presentation of Ethical Appreciation (CAAE) numbers 30204620.4.2001.5327 and 55385322.9.1001.0086. Data extracted from hospital records were stored in a deidentified format.

### 2.4. Data Collection

The researchers retrospectively collected deidentified data from individual medical records using the Research Electronic Data Capture (REDCap) [17]. Three different forms were employed: (i) an enrollment form that encompassed baseline characteristics, medical and obstetrical history, SARS-CoV-2 exposure, and COVID-19 vaccine information; (ii) COVID-19 follow-up forms, which documented information on infection natural history; and (iii) a pregnancy follow-up form, which was completed at the end of pregnancy which contained data on pregnancy and neonatal outcomes. These forms were developed within the framework of the COVI-Preg registry, which aimed to assess the impact of SARS-CoV-2 infection on pregnant women and their foetuses/newborns, as previously described by Panchaud et al. [18].

### 2.5. Participants

Pregnant women attending hospitals for obstetric reasons (including regular prenatal appointments and pregnancy complications) or those presenting COVID-19 symptoms were included in the study. Eligibility criteria were (i) being older than 18 years and (ii) testing positive for SARS-CoV-2 during pregnancy. The tests used to determine SARS-CoV-2 status included nasopharyngeal reverse transcriptase polymerase chain reaction (RT-PCR) and lateral flow immunoassay for detection of antigen tests. During the study period, except for the first 5 months, a COVID-19 test was compulsory for every pregnant person admitted to hospital, as well as routine repeat tests for those who spent more than a week in hospital. Even pregnant women admitted to hospital for obstetric reasons who did not show symptoms of COVID-19 had to be tested. If an individual suspected with COVID-19 initially obtained a negative result for COVID-19 test, the test was repeated later, between the 2nd and 7th day after the onset of symptoms. Pregnant women with missing information regarding the date of COVID-19 diagnosis or the type of test were excluded, as well as those with a lack of information to estimate the last menstrual period. Exclusion criteria were applied after data collection and before statistical analysis step.

### 2.6. COVID-19 Clinical Symptoms and Severity

Information about COVID-19 included clinical manifestations, date and result of the test, vaccination, and progression of infection, as registered in the hospital records.

The following clinical symptoms from the hospital records were collected: fever defined as body temperature greater than 37°C, cough, dyspnoea, sore throat, myalgia, fatigue, headache, nausea and/or vomiting, sputum production, anosmia and/or ageusia. In the absence of symptoms during the clinical evolution of the disease, the case was assigned as asymptomatic.

The severity of COVID-19 was classified into two groups: pregnant women who needed inpatient management due to COVID-19 (moderate, severe, and critical cases) were defined as hospitalized, and those who did not require admission were defined as outpatient. The latter consisted of pregnant women who were asymptomatic or had COVID-19 symptoms that did not require any treatment as well as pregnant women who were at the hospital due to obstetrics reasons (e.g., delivery).

### 2.7. Exposure to Medications and Guidelines to Treat COVID-19

We recorded the use of any medication around the time of COVID-19 diagnosis as self-reported or prescribed in the hospital, in the 3 days before the diagnosis of COVID-19, as extracted from the hospital records. For the self-reported use, pregnant women were asked if they use any medication in previous days, without presenting any list of options. Our definition was irrespective of whether the medication treated chronic conditions, managed symptoms, or treated COVID-19 infection, no matter the dose or duration. Iron, mineral supplements, vitamins, herbal products, and any type of complementary medicine are not counted as medications. The medications' names were registered in REDCap and grouped according to ATC-level 2 codes. We also carried out a manual search and consulted specialists to identify guidelines for the treatment of COVID-19 among pregnant women, in order to systematize the changes of recommendations regarding the use of medications for the treatment of the infection. The recommendations are presented in accordance with a grading system similar to that used in a previous study [19].

### 2.8. Covariates and Measures

Information about covariates was extracted from routine hospital records. The information included sociodemographic characteristics (age, educational level, and marital status), health and diseases (chronic conditions), and obstetrical factors (date of last menstrual period, nulliparity, number of foetuses in the currency pregnancy, and obstetrics conditions). Covariates were categorized as presented in Table 1.

The gestational age at infection was calculated using the date of the onset of symptoms (or the date of the test) and the date of the last menstrual period or through ultrasound. Trimesters were defined according to the American College of Obstetricians and Gynecologists classification [20].

### 2.9. Statistical Analysis

Descriptive statistics were used to present sociodemographic, obstetrical, and COVID-19 characteristics. To estimate the prevalence of medication use, we fit generalized linear mixed models in which the hospital of origin was the random effect and the prevalence of medication use for each hospital was the dependent variable. We evaluated the medication use by trimester and by COVID-19 severity. The crude prevalence by group (trimesters or COVID-19 severity) was calculated as the proportion of pregnant women in each group exposed to at least one medication divided by the total number of pregnant women in the same group.

To identify predictors for COVID-19 hospitalization, we performed univariate and multivariate analyses using logistic regression with a random effect for the hospital of origin. We conducted a purposeful selection of covariates, as described by Hosmer et al. [21]. First, candidate covariates with a *p* value less than 0.25 in the univariate analyses were selected and included in the multivariate model. Then, variables with a *p* value greater than 0.1 were removed one by one, and we assessed the change in the effect estimates of the retained variables. If the latter change was less than 20%, the variable was removed from the multivariate reduced model. Then, we evaluated if adding individually each previously excluded variable could have a *p* value < 0.1 or change the estimates by more than 20% compared with the reduced model. After that, we reach the final model. We conducted a sensitivity analysis by restricting the data to pregnant women with confirmed COVID-19 through RT-PCR tests. Statistical analyses were performed using R. A *p* value less than 0.05 was considered statistically significant.

We conducted multiple imputations of missing data on covariates using logistic regression implemented by the mice package v. 3.15.0, setting 100 imputations for each missing value [22]. The variables with missing values were educational level, marital status, gravidity, and number of foetuses. Their proportions are presented in Table 1. The following variables were input as predictive variables for the imputation model: outcome of interest (hospitalized due to COVID-19 or outpatient), maternal age, pre- or gestational hypertensive disorders, pre- or gestational diabetes, pre-existing pulmonary, cardiac, or renal conditions, trimester of infection, the status of COVID-19 vaccination, and hospital of origin.

## 3. Results

**Figure 1:**
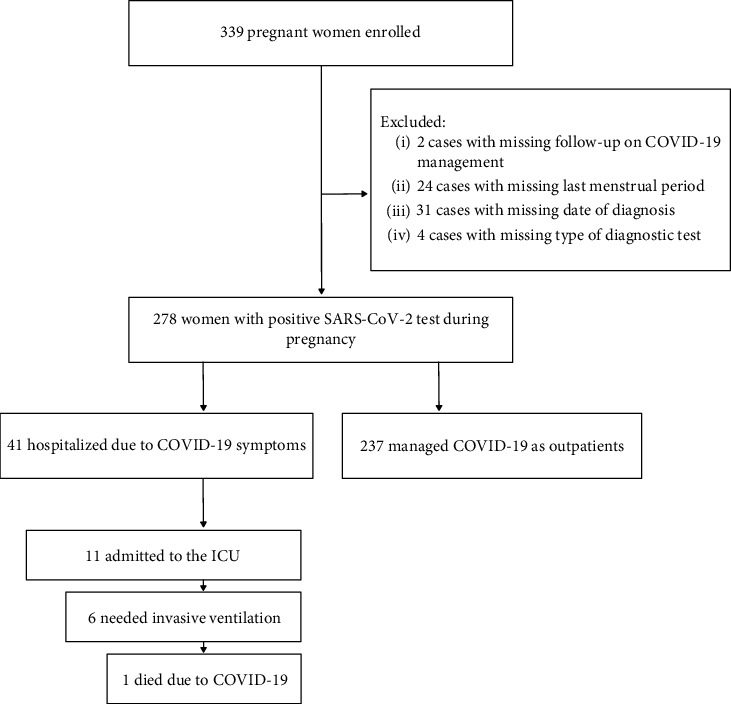
Flowchart of the process for enrollment, selection, and classification of participants diagnosed with COVID-19 during pregnancy.

From March 8, 2020, to August 31, 2022, we identified 339 pregnant women who attended the two hospitals and received a COVID-19 diagnosis (Figure 1). Among these cases, we excluded two due to missing follow-up information related to COVID-19 management. Additionally, after data collection, we excluded 24 cases due to insufficient information about the last menstrual period, 31 cases with inadequate data regarding the date of diagnosis, and four cases lacking information about the type of test performed for diagnosis. As a result, 278 women with a positive SARS-CoV-2 test during pregnancy were included in this study, 41 of whom needed hospitalization for COVID-19 treatment.

The median age of pregnant women was 31 years (interquartile (IQR) 27, 36 years), and 25.2% (*n* = 70) were older than 35 years at the time of SARS-CoV-2 infection diagnosis (Table 1). Almost half of the women had a university degree (*n* = 130; 46.8%), and the majority were in a married/committed relationship (*n* = 208, 74.8%). The most common pre-existing medical conditions were thyroid imbalance (*n* = 19, 6.8%) and hypertension (*n* = 14, 5.0%). The most common obstetrics conditions were gestational diabetes (*n* = 39, 14.0%) and gestational hypertension (*n* = 20, 7.2%) (Table 1).

SARS-CoV-2 infections were most frequent in the third trimester of pregnancy (*n* = 149, 53.6%). The most reported symptoms were cough (*n* = 131, 47.1%), fever (*n* = 110, 39.6%), myalgia (*n* = 102, 39.6%), and headache (*n* = 102, 36.7%). Only 19 women were asymptomatic (6.8%). Approximately half (*n* = 138, 49.6%) of the pregnant women had not received any vaccine dose against SARS-CoV-2 at the time of the infection. Asymptomatic to mild COVID-19 was more common (*n* = 237, 85.3%). Forty-one (14.7%) women were hospitalized to treat COVID-19, of whom 11 (4.0%) required admissions to the ICU, and six (2.2%) needed invasive ventilation. One woman died due to COVID-19 complications (Table 2).

### 3.1. Use of Medications

The use of at least one medication at the time of infection was 47.5%, 32.6%, and 55.2%, among women at the first, second, and third trimesters of gestation, respectively (Table 3). Analgesics (range 2.4% to 20.0%), antibacterials (range 15.0% to 23.1%), and corticosteroids (range 7.2% to 10.4%) were the medications with higher prevalence in all the trimesters (Table 3). Drugs for thyroid therapy had a high prevalence among women in the second pregnancy trimester (10.1%, 95% CI 5.3, 18.3), particularly, reflecting the underlying medical conditions of the population studied. Among pregnant women infected in the third trimester, a high prevalence of the use of antibacterials (23.1%, 95% CI 6.1, 58.1), corticosteroids (10.4%, 95% CI 5.4, 19.0), and antivirals (8.0%, 95% CI 4.3, 14.2) was observed.

The proportions and corresponding 95% confidence intervals (CIs) were estimated by generalized linear mixed models with random effects on the hospital of origin.

Table 4 presents the prevalence of medication uses for hospitalized and outpatient pregnant women. Antibacterials were the most common medication among the hospitalized group (62.7%, 95% CI 29.8, 87.0) and the outpatient group (8.1%, 95% CI 5.2, 12.2). Following antibacterials, corticosteroids (27.0%, 95% CI 10.0, 55.2) and antithrombotic agents (12.2%, 95% CI 5.2, 26.1) were the medication more utilized among hospitalized pregnant women, while in the outpatient group were thyroid therapy agents (6.3%, 95% CI 3.9, 10.2), antivirals (4.6%, 95% CI 2.6, 8.2), and analgesics (3.8%, 95% CI 0.2, 51.0). The overall prevalence of at least one medication use was higher in the hospitalized group (74.4%, 95% CI 34.9, 94.1) compared to the outpatient group (35.9%, 95% CI 30.0, 42.2). Table S1 summarizes medication recommendations for the treatment of COVID-19 among pregnant women during the study period, based on the guidelines of Brazilian specialized authorities.

### 3.2. Risk Factors for Hospitalization Due to COVID-19

In univariate analysis, sociodemographic, health and obstetric characteristics, and vaccination against COVID-19 were all associated with hospitalization due to COVID-19 (Table 5). In the multivariable reduced model, only two factors remained strong predictors of maternal hospitalization. Having pre- or gestational hypertensive disorder was associated with almost five-fold increased odds of hospitalization relative to not having the disorder (aOR 4.94, 95% CI 1.64, 14.87). COVID-19 vaccination (at least one dose) was associated with 87% reduced odds for hospitalization (aOR 0.13, 95% CI 0.04, 0.39) compared to being unvaccinated. When assessing data exclusively from pregnant women diagnosed with COVID-19 by RT-PCR, sociodemographic, health, and obstetric characteristics were once again associated with hospitalization due to COVID-19 in the univariate analysis (Table S2). After selecting predictors for hospitalization, the final model retained only one predictor: receiving at least one dose of the COVID-19 vaccine (OR 0.11, 95% CI 0.02, 0.54).

## 4. Discussion

This study reports new information about medication use among pregnant women diagnosed with SARS-CoV-2 infection. Additionally, this study highlights the risk factors for hospitalization due to COVID-19, specifically from two Brazilian tertiary care centres. Brazil is a country of continental proportions, marked by social and economic inequalities that manifest in disparities in access to maternal health care [23, 24]. A Brazilian study based on 2020 data from the national surveillance system for severe acute respiratory syndrome among pregnant and postpartum women diagnosed with COVID-19 pointed out a lack of access to health care among those who died [25]. Another study demonstrated excess maternal mortality in 2020 [5]. Since the first year of the pandemic, experts have drawn attention to the tragedy unfolding due to the impact of COVID-19 on maternal mortality in Brazil [26–28]. By December 2022, more than 24,000 pregnant and postpartum women were infected with SARS-CoV-2 in Brazil, of which 2000 of these women died [29]. As COVID-19 is a risk factor for adverse outcomes among pregnant women [1], understanding the clinical characteristics of infection in this population is a crucial task to delineate specific treatment strategies and ensure that these strategies are informed by the latest evidence. The present study incorporates information about COVID-19 during pregnancy in vaccinated and unvaccinated women across different waves of the pandemic. During the COVID-19 pandemic, for patients admitted to obstetric centres, screening for SARS-CoV-2 was mandatory. Therefore, pregnant women admitted to the hospital due to obstetric reasons could have been diagnosed with SARS-CoV-2 infection even if they were asymptomatic or presented symptoms at a later time.

In line with the results of other studies [30, 31], we found that more than half of the pregnant women included in this study were infected by SARS-CoV-2 in the third trimester of gestation. Anatomical and physiological changes become more prominent as pregnancy advances [32, 33] and can make respiratory symptoms more severe. Consequently, women in more advanced pregnancies possibly have more noticeable symptoms and seek medical care more easily as a precaution than those in early pregnancy. Moreover, factors related to gestational age, such as diabetes and gestational hypertension, are responsible for increasing the chances of a pregnant woman being admitted to an obstetric centre, triggering a higher proportion of pregnant women at the end of pregnancy being observed in hospital-based studies. In addition, immunological changes that occur as pregnancy progresses may increase vulnerability to infections [34, 35]. However, this trend is not evident with all other types of infection. For example, the need for hospitalization for the treatment of influenza infection does not differ between trimesters [36, 37]. Despite the aforementioned difference between trimesters, Leung et al. [4], found that 62.3% of pregnant women hospitalized with COVID-19 were in their third trimester of pregnancy. The authors' results show, after adjusting for other comorbidity factors, that women in a later gestational period were not at a higher risk of COVID-19-related morbidity or mortality than women in earlier trimesters.

Antibacterials were the most commonly used medication among pregnant women. There is no consensus about the use of antibiotics in Brazilian guidelines regarding COVID-19 in pregnancy management (see Table S1). While some guidelines recommend antibiotics for secondary bacterial infection, another one recommends the use of azithromycin for pregnant women at all COVID-19 severity levels, independent of signs of bacterial infection. At the beginning of the pandemic, azithromycin was postulated as a potential drug to treat COVID-19 in combination with hydroxychloroquine but later was demonstrated to be ineffective [38, 39]. However, the COVID-19 pandemic in Brazil has been characterized not only by a lack of coordination in actions to combat the spread of the virus [40, 41], but also by the promotion of antiscience practices [42–45], such as the recommendation for the prophylactic use of medications to prevent the worsening of the disease, despite growing evidence that these medications were not effective for COVID-19 [46].

Recently, a systematic review with meta-analysis found a slight but significant association between prenatal exposure to azithromycin and major congenital anomalies compared to prenatal exposure to other antibiotics [47]. This finding should be taken carefully as it could be due to maternal confounders. Although the current study observes a substantial difference in the prevalence rate of the antibacterials used between hospitalized and outpatient pregnant women, the prevalence in the outpatient group was higher than the prevalence observed in previous studies about medication among pregnant women during the COVID-19 pandemic [48, 49].

Analgesics are recommended for managing COVID-19 symptoms, and as expected, in the present study, they had a higher use prevalence among outpatient pregnant women. Corticosteroids had an overall high prevalence too, particularly among the hospitalized group. This medication is recommended to treat patients with severe COVID-19 [50], but, in the case of pregnant women, corticosteroids can be useful for foetal pulmonary maturation in cases with a risk of prematurity. The prophylactic use of antithrombotics is recommended due to the risk of coagulopathy, especially for pregnant women with severe COVID-19 [51, 52]. Among the current study's participants, antithrombotics were prevalent among women with more advanced gestational ages and who present with moderate-to-severe COVID-19. The overall prevalence rate of antithrombotics observed in the current study is close to that reported in one previous study [53], but lower than other similar studies [48, 49].

We have identified only two potential predictors for hospitalization due to COVID-19: pre- or gestational hypertension and COVID-19 vaccination, both of which are in line with prior works. In a Canadian study of SARS-CoV-2 infection during pregnancy, pregnant women with pre-existing hypertension had a 2.4-fold increase in the risk for hospitalization and a 3.5-fold increase in the risk for ICU admission [54]. In the study of Galang et al. [55] with data from the United Kingdom's Obstetric Surveillance System, pregnant patients with chronic hypertension, but not those with gestational hypertension, had a 1.4-fold increase in the risk for moderate-to-severe or critical COVID-19. In the results of the sensitivity analysis, pre-existing or gestational hypertensive disorders were not included in the final model of prediction to hospitalization due to COVID-19, despite being a strong predictor in the main analysis. While other prior studies have utilized a larger number of predictors [31], due to limitations posed by the number of participants, certain predictors were not found to be statistically significant when specifying the models used in the current study, including being over 35 years of age, having pre- or gestational diabetes, and having pre- or gestational hypertensive disorders, the latter strictly in the sensitivity analysis. Predictors operationalized in other studies highlight the influence of comorbidities on the severity of COVID-19 infection. Galang et al. [55] found that obesity, chronic lung disease, chronic hypertension, and diabetes mellitus are risk factors for moderate-to-severe or critical COVID-19 illness, and the number of underlying medical or obstetric-related conditions has a positive correlation with the severity of COVID-19.

We observed that 90.2% of the pregnant women that needed hospitalization to treat COVID-19 were unvaccinated. On a related note, we also found that being vaccinated against COVID-19 was a strong protective factor against hospitalization for participants that had been diagnosed with COVID-19, with a consistent effect size observed in both the main and sensitivity analyses. Although previous researchers have addressed initial concerns about the safety of the COVID-19 vaccination for pregnant women [56, 57], the cumulative evidence shows that COVID-19 vaccines during pregnancy do not increase the risk of adverse outcomes. Instead, the vaccine reduces the rates of emergency C-sections [58], stillbirth [59, 60], premature birth [59, 60], and neonatal intensive care unit admission [58, 60]. The COVID-19 vaccination among pregnant women should be promoted, and the cumulative research behind COVID-19 vaccinations should guide the protocols for the vaccination of pregnant women.

A limited part of the study population, 41 out of 278 individuals, was admitted to hospitals for the management of COVID-19, comprising the hospitalized group. In a comparable study on COVID-19 during pregnancy, 20.9% of participants developed moderate-to-severe or critical COVID-19 severity [55]. Another study reported that 78% of pregnant women developed mild or asymptomatic illness, while 22% experienced moderate to severe [61]. Both studies referenced had their study period preceding the availability of vaccines for pregnant women. While the World Health Organization delineates four severity groups [62], in addition to the asymptomatic group, we choose to categorize the study population based on the trajectory of illness management, whether individuals were or were not hospitalized for COVID-19 treatment. Within the hospitalized group, a minority developed severe or critical COVID-19, while the majority presented moderate severity. This suggests that hospitalization per se may not indicate a negative adverse effect. However, noteworthy differences in the prevalence of medication use between the two groups in our study suggest that moderate, severe, and critical levels may, to some extent, have a greater risk of drug exposure during pregnancy compared to the outpatient group. Nevertheless, the composition of the hospitalized group in this study may have attenuated differences in estimating risk factors for hospitalization. Future studies with better resolution between different levels of severity will be able to estimate risk factors more assertively. Additionally, an important knowledge gap to be addressed is understanding at what level hospitalization to treat COVID-19 may pose a greater risk for obstetric and neonatal outcomes, as we did not assess these outcomes.

Our study has some limitations. The first of these limitations relates to the study population. Besides the limited number of participants as compared to other studies, the population enrolled in this study was a convenience sample from two hospitals, wherein the women were admitted for obstetrics or for the treatment of COVID-19. As a result, the sample may be susceptible to selection bias. Initially, during the early months of the COVID-19 pandemic in Brazil, testing was limited to individuals presenting symptoms, leading to the exclusion of asymptomatic cases from our study during this period and, therefore, underestimating the cases. However, later in the recruitment period, a screening protocol was implemented for all pregnant women admitted to hospitals, aiming to capture asymptomatic cases as well. The overall rate of asymptomatic cases in our sample was 6.8%. Furthermore, our study spans a timeframe that includes different variant strains of SARS-CoV-2 and encountered a period with no COVID-19 vaccine existence in the initial months. These factors likely change in the prevalence of asymptomatic cases over time. Additionally, the demographic characteristics of patients across both hospitals differed, including age, education level, and socioeconomic status. The current study's population is, on average, older and has a higher level of education than the general population of pregnant women in Brazil [63]. Furthermore, COVID-19 testing protocols changed over time, which impacted recruitment and identification rates of severe cases. Similarly, another change was the availability of the vaccine from 2021 onwards. As a result, the incidence of more severe cases of COVID-19 has decreased, and the results of this study need to be interpreted considering these changes. Another key limitation is that the limited population size in estimating medication usage resulted in wide CIs, which may indicate inaccurate estimates. To overcome this limitation, the statistical analyses were replanned, and the comparisons made through the study's analyses were revised to account for the limited number of observations. However, any generalizations inferred from the results of this study should take into account the limited sample.

The final limitation of this study is that the data extraction process was performed from electronic hospital records. This is an issue because the data were input by a variety of healthcare professionals, so there is no standardized protocol for registering patients. This results in missing information in some patient records. The missing data dimension corresponds to up to 6% for variables at the population level and up to 12% in subgroups. Multiple imputations were performed to manage the missing data in the univariate and multivariate analyses. The records about medications, except for the use of drugs for chronic conditions, represent the use during a hospital stay or the days leading up to a hospital stay (as some women take medications when first experiencing symptoms before going to a hospital) and cannot be interpreted to correspond to the entire gestation period. Additionally, we established only two groups for comparison, categorized by the clinical manifestation and treatment for COVID-19 documented in the hospital records. In the hospitalized group, a minority experienced a critical severity (26.8%), and the remaining portion may have mitigated the estimates in relation to the outpatient group. However, it was still possible to estimate predictors for hospitalization and identify differences in the prevalence of medication use between the two groups. Although the study took place over an extended period of time, we did not explore differences in clinical manifestations, outcomes, and medication use across each wave of the COVID-19 pandemic. On the other hand, this study was able to incorporate cases of COVID-19 over a long period of time, and moreover, the study includes participants with a variety of vaccination statuses.

## 5. Conclusions

In this study among SARS-CoV-2-positive pregnant women from two Brazilian hospitals, the most frequently used medications around the time of infection were antibacterials, analgesics, and corticosteroids. Among pregnant women hospitalized to treat COVID-19, a high prevalence of antibacterials and corticosteroids was observed. Women with pre- and gestational hypertensive disorders have almost a five-fold increased likelihood of hospitalization during pregnancy due to COVID-19, despite this result not being consistent in the sensitivity analysis. Vaccination was the strongest protective factor against hospital admission due to COVID-19. The COVID-19 vaccination among pregnant women should be promoted, and pregnant women diagnosed with COVID-19 who have hypertensive disorders should be closely monitored.

## Figures and Tables

**Table 1:** Sociodemographic and obstetrics characteristics of the pregnant women with positive SARS-CoV-2 test.

**Characteristic**	**Severity**		
	**Hospitalized** **(** ***N*** ** = 41)** **n** **(%)**	**Outpatient** **(** ***N*** ** = 237)** **n** **(%)**	**Total** **(** ***N*** ** = 278)** **n** **(%)**
Hospital			
HCPA	30 (73.2)	32 (13.5)	62 (22.3)
SEPACO	11 (26.8)	205 (86.5)	216 (77.7)
Age			
Median (IQR)	31 (25 – 37)	30 (27–35)	31 (27–36)
Age > 35 years	16 (39.0)	54 (22.8)	70 (25.2)
Educational level			
University	8 (19.5)	122 (51.5)	130 (46.8)
High school/primary school	31 (75.6)	106 (44.7)	137 (49.3)
Missing	2 (4.9)	9 (3.8)	11 (3.9)
Marital status			
Married/committed relationship	28 (68.3)	180 (75.9)	208 (74.8)
Single or divorced	8 (19.5)	45 (19.0)	53 (19.1)
Missing	5 (12.2)	12 (5.0)	17 (6.1)
Medical pre-existing conditions			
Thyroid imbalance	3 (7.3)	16 (6.8)	19 (6.8)
Hypertension	6 (14.6)	8 (3.4)	14 (5.0)
Pulmonary	3 (7.3)	5 (2.1)	8 (2.9)
Diabetes	3 (7.3)	2 (0.8)	5 (1.8)
Cardiac	0 (0)	2 (0.8)	2 (0.7)
Others (neurologic, digestive, renal, urological, hematologic, and autoimmune)	9 (22.0)	23 (9.7)	32 (11.5)
Obstetric conditions			
Gestational diabetes	8 (19.5)	31 (13.1)	39 (14.0)
Gestational hypertension	7 (17.1)	13 (5.5)	20 (7.2)
Previous pregnancies			
Multiparous	25 (61.0)	115 (48.5)	140 (50.4)
Nulliparous	15 (36.6)	119 (50.2)	134 (48.2)
Missing	1 (2.4)	3 (1.3)	4 (1.4)
Number of foetuses			
1	39 (95.1)	229 (96.6)	268 (96.4)
2	1 (2.4)	7 (3.0)	8 (2.9)
Missing	1 (2.4)	1 (0.4)	2 (0.7)

Abbreviations: HCPA = Hospital de Clínicas de Porto Alegre; IQR = interquartile; SEPACO = SEPACO Hospital e Maternidade.

**Table 2:** Clinical characteristics related to COVID-19 among pregnant women.

**COVID-19 characteristics**	**Severity**		
	**Hospitalized** **(** ***N*** ** = 41)** **n** **(%)**	**Outpatient** **(** ***N*** ** = 237)** **n** **(%)**	**Total** **(** ***N*** ** = 278)** **n** **(%)**
Type of diagnostic test			
RT-PCR	39 (95.1)	181 (76.4)	220 (79.1)
Antigen immunoassay	2 (4.9)	56 (23.6)	58 (20.9)
Trimester of the diagnosis			
First	2 (4.9)	38 (16.0)	40 (14.4)
Second	10 (24.4)	79 (33.3)	89 (32.0)
Third	29 (70.7)	120 (50.6)	149 (53.6)
Clinical manifestations			
Cough	22 (53.7)	109 (46.0)	131 (47.1)
Fever	23 (56.1)	87 (36.7)	110 (39.6)
Myalgia	12 (29.3)	98 (41.4)	110 (39.6)
Headache	8 (19.5)	94 (39.7)	102 (36.7)
Sputum production	8 (19.5)	77 (32.5)	85 (30.6)
Anosmia and/or ageusia	10 (24.4)	66 (27.8)	76 (27.3)
Sore throat	7 (17.1)	64 (27.0)	71 (25.5)
Dyspnea	28 (68.3)	37 (15.6)	65 (23.4)
Fatigue	7 (17.1)	62 (26.2)	69 (24.8)
Nausea and/or vomiting	8 (19.5)	30 (12.7)	38 (13.7)
Asymptomatic	0 (0)	19 (8.0)	19 (6.8)
Vaccine against SARS-CoV-2 before infection			
Unvaccinated	37 (90.2)	101 (42.6)	138 (49.6)
1 dose	1 (2.4)	19 (8.0)	20 (7.2)
2 doses	2 (4.9)	94 (39.7)	96 (34.5)
3 doses	1 (2.4)	23 (9.7)	24 (8.6)
Severe adverse outcomes			
Admission to ICU	11 (26.8)	—	11 (4.0)
Invasive ventilation	6 (14.6)	—	6 (2.2)
Death	1 (2.4)	—	1 (0.4)

**Table 3:** Prevalence of the most common medications used among pregnant women according to trimester of infection.

**Group of medications (ATC-level 2 codes)**	**Trimester of diagnosis**					
	**First** **(** ***N*** ** = 40)**		**Second** **(** ***N*** ** = 89)**		**Third** **(** ***N*** ** = 149)**	
	**n** **(%)**	**% (95% CI)**	**n** **(%)**	**% (95% CI)**	**n** **(%)**	**% (95% CI)**
Analgesics (N02)	8 (20.0)	9.1–35.6	5 (5.6)	2.4–12.8	16 (2.4)	0.1–79.2
Antibacterials (J01)	6 (15.0)	5.7–29.8	11 (16.8)	4.8–44.7	30 (23.1)	6.1–58.1
Corticosteroids (H02)	3 (7.5)	1.6–20.0	5 (7.2)	1.4–29.7	13 (10.4)	5.4–19.0
Thyroid therapy (H03)	2 (5.0)	0.6–16.9	9 (10.1)	5.3–18.3	5 (4.0)	1.7–9.3
Antivirals (J05)	2 (5.0)	0.6–16.9	2 (2.2)	0.6–8.5	10 (8.0)	4.3–14.2
Antihistamines (R06)	2 (5.0)	0.6–16.9	1 (1.1)	0.1–7.5	6 (2.5)	0.1–30.2
Antihypertensives (C02, C03, C04, C07, C08, and/or C09)	1 (2.5)	0.1–13.2	2 (2.2)	0.6–8.5	8 (6.4)	3.2–12.3
Antithrombotic agents (B01)	—	—	2 (2.2)	0.6–8.5	5 (3.7)	1.1–12.3
Medicines for obstructive airway disease (R03)	—	—	2 (2.2)	0.6–8.5	5 (3.7)	1.1–12.3
At least one medication	19 (47.5)	31.5–63.9	29 (32.6)	23.7–42.9	69 (55.2)	46.4–63.7

*Note:* The proportions and corresponding 95% CIs were estimated by generalized linear mixed models with random effects on the hospital of origin.

**Table 4:** Prevalence of the most common medication used among pregnant women in the hospitalized and nonhospitalized groups.

**Group of medications (ATC-level 2 codes)**	**Hospitalized** **(** ***N*** ** = 41)**		**Outpatient** **(** ***N*** ** = 237)**		**Total** **(** ***N*** ** = 278)**	
	**n** **(%)**	**95% CI**	**n** **(%)**	**95% CI**	**n** **(%)**	**95% CI**
Antibacterials (J01)	28 (62.7)	29.8–87.0	19 (8.1)	5.2–12.2	47 (21.7)	5.8–55.3
Corticosteroids (H02)	12 (27.0)	10.0–55.2	9 (3.8)	2.0–7.1	21 (8.9)	3.4–21.6
Antithrombotic agents (B01)	5 (12.2)	5.2–26.1	2 (0.8)	0.2–3.3	7 (2.7)	0.9–7.9
Antihypertensives (C02, C03, C04, C07, C08, and/or C09)	4 (9.8)	3.7–23.3	7 (3.0)	1.4–6.1	11 (3.9)	2.2–7.0
Medicines for obstructive airway disease (R03)	4 (9.8)	3.7–23.3	3 (1.3)	0.4–3.8	7 (2.7)	0.9–7.9
Antivirals (J05)	3 (7.3)	2.4–20.4	11 (4.6)	2.6–8.2	14 (5.0)	3.0–8.3
Analgesics (N02)	2 (4.9)	1.2–17.5	27 (3.8)	0.2–51.0	29 (5.9)	1.3–22.8
Thyroid therapy (H03)	1 (2.4)	0.3–15.4	15 (6.3)	3.9–10.2	16 (5.8)	3.6–9.2
Antihistamines (R06)	—	—	9 (3.8)	2.0–7.1	9 (3.2)	1.7–6.1
At least one medication	32 (74.4)	34.9–94.1	85 (35.9)	30.0–42.2	117 (44.8)	33.7–56.3

*Note*: The proportions and corresponding 95% CIs were estimated by generalized linear mixed models with random effects on the hospital of origin.

**Table 5:** Risk factor estimates for hospitalization due to COVID-19. Multivariable analysis was performed after the selection of significant variables for the model.

**Risk factor**	**Univariate analysis** **(** ***N*** ** = 278** **^a^** **)**		**Full model multivariable analysis** **(** ***N*** ** = 278** **^a^** **)**		**Reduced model multivariable analysis** **(** ***N*** ** = 278** **^a^** **)**	
	**OR**	**95% CI**	**OR**	**95% CI**	**OR**	**95% CI**
At least 1 dose of COVID-19 vaccine	0.12	0.04–0.37	0.13	0.04–0.39	0.13	0.04–0.39
Pre- or gestational hypertensive disorders	5.47	1.92–15.55	3.88	1.17–12.94	4.94	1.65–14.87
Maternal age > 35 years	2.58	1.12–5.95	1.94	0.74–5.08		
Pre- or gestational diabetes	2.14	0.84–5.42	1.42	0.47–4.38		
Gestational age at infection > 196 days	1.08	0.46–2.54				
Pre-existing pulmonary, cardiac, and renal conditions	1.11	0.28–4.43				
Married/committed relationship	1.02	0.39–2.66				
Nulliparity	0.86	0.39–1.89				
University degree	0.55	0.21–1.41	0.82	0.29–2.31		
Multiple pregnancies	0.45	0.04–4.64				

*Note:* The full model includes all significant covariates from the univariate analyses (*p* value < 0.25). The reduced model includes all variables that remain after the selection process (*p* value < 0.1 or change in other estimates > 20%). The reference category is “No” for all risk factors, unless otherwise stated. For marital status, single, never married or divorced, was the reference group. For education, high school/primary school was the reference group.

^a^Estimates were computed under multiple imputations to missing data.

## Data Availability

The deidentified data and the analysis codes of this study are available upon request to Ricardo Rohweder at ricardo.rohweder@ufrgs.br once justification for access is presented.

## Supporting Information

Supporting information Additional supporting information can be found online in the Supporting Information section. Table S1 Brazilian guidelines on the medicines used to treat COVID-19 in pregnancy. The recommendations for usage in each guideline are denoted by category and by specific drugs. Alterations over time within the same period of the study are indicated using a grading system. Table S2 Risk factor estimates for hospitalization due to COVID-19 exclusively considering cases diagnosed by polymerase chain reaction (PCR). Multivariable analysis was performed after the selection of significant variables for the model.
