# Supplementary material for: Medication Use Among Pregnant Women With SARS-CoV-2 Infection and Risk of Hospitalization—A Study in Two Brazilian Hospitals
Source: J Pregnancy. 2024 Jun 6;2024:8915166. doi: 10.1155/2024/8915166 (PMC11254464; doi:10.1155/2024/8915166)
Supplement: Supporting information — Additional supporting information can be found online in the Supporting Information section. Table S1 Brazilian guidelines on the medicines used to treat COVID-19 in pregnancy. The recommendations for usage in each guideline are denoted by category and by specific drugs. Alterations over time within the same period of the study are indicated using a grading system. Table S2 Risk factor estimates for hospitalization due to COVID-19 exclusively considering cases diagnosed by polymerase chain reaction (PCR). Multivariable analysis was performed after the selection of significant variables for the model. [file 8915166.f1.docx]

Supplementary Table 1 - Brazilian guidelines on the medicines used to treat COVID-19 in pregnancy.

| Guideline | Category | Drug | 2020 | | | | | | | | | | 2021 | | | | | | | | | | | | 2022 |
| --- | --- | --- | --- | --- | --- | --- | --- | --- | --- | --- | --- | --- | --- | --- | --- | --- | --- | --- | --- | --- | --- | --- | --- | --- | --- |
|  |  |  | Mar | Apr | May | Jun | Jul | Aug | Sep | Oct | Nov | Dec | Jan | Feb | Mar | Apr | May | Jun | Jul | Aug | Sep | Oct | Nov | Dec |  |
| MRAGP^1,2^ | Analgesics |  |  |  |  |  |  |  |  |  |  |  |  |  |  |  |  |  |  |  |  |  |  |  |  |
|  | Antiviral | Oseltamivir** |  |  |  |  |  |  |  |  |  |  |  |  |  |  |  |  |  |  |  |  |  |  |  |
|  | Antibiotics | Ceftriaxone |  |  |  |  |  |  |  |  |  |  |  |  |  |  |  |  |  |  |  |  |  |  |  |
|  |  | Azithromycin |  |  |  |  |  |  |  |  |  |  |  |  |  |  |  |  |  |  |  |  |  |  |  |
|  |  | Amoxicillin + Clavulanate |  |  |  |  |  |  |  |  |  |  |  |  |  |  |  |  |  |  |  |  |  |  |  |
|  | Antithrombotic | Heparin*** |  |  |  |  |  |  |  |  |  |  |  |  |  |  |  |  |  |  |  |  |  |  |  |
|  |  | Enoxaparin*** |  |  |  |  |  |  |  |  |  |  |  |  |  |  |  |  |  |  |  |  |  |  |  |
|  | Corticosteroids | Metilprednisolone**** |  |  |  |  |  |  |  |  |  |  |  |  |  |  |  |  |  |  |  |  |  |  |  |
|  |  | Dexamethasone**** |  |  |  |  |  |  |  |  |  |  |  |  |  |  |  |  |  |  |  |  |  |  |  |
| NI^3,4,5^ | Antimalarials | Chloroquine |  |  |  |  |  |  |  |  |  |  |  |  |  |  |  |  |  |  |  |  |  |  |  |
|  |  | Hydroxychloroquine |  |  |  |  |  |  |  |  |  |  |  |  |  |  |  |  |  |  |  |  |  |  |  |
|  | Antibiotics | Azithromycin |  |  |  |  |  |  |  |  |  |  |  |  |  |  |  |  |  |  |  |  |  |  |  |
| PAPPA^6,7^ | Antiviral | Oseltamivir***** |  |  |  |  |  |  |  |  |  |  |  |  |  |  |  |  |  |  |  |  |  |  |  |
|  |  | Others |  |  |  |  |  |  |  |  |  |  |  |  |  |  |  |  |  |  |  |  |  |  |  |
|  | Antibiotics |  |  |  |  |  |  |  |  |  |  |  |  |  |  |  |  |  |  |  |  |  |  |  |  |
|  | Corticosteroids |  |  |  |  |  |  |  |  |  |  |  |  |  |  |  |  |  |  |  |  |  |  |  |  |
|  | Convalescent plasma |  |  |  |  |  |  |  |  |  |  |  |  |  |  |  |  |  |  |  |  |  |  |  |  |
|  | Antiprotozoals | Hydroxychloroquine |  |  |  |  |  |  |  |  |  |  |  |  |  |  |  |  |  |  |  |  |  |  |  |
|  | Anthelmintics |  |  |  |  |  |  |  |  |  |  |  |  |  |  |  |  |  |  |  |  |  |  |  |  |
|  | Antithrombotic | Heparin****** |  |  |  |  |  |  |  |  |  |  |  |  |  |  |  |  |  |  |  |  |  |  |  |

MRAGP: Manual de Recomendações para a Assistência à Gestante e Puérpera frente à Pandemia de COVID-19;

NI: Nota Informativa com Orientações para manuseio medicamentoso precoce de pacientes com diagnóstico da COVID-19;

PAPPA: Protocolo de atendimento no parto, puerpério e abortamento durante a pandemia de COVID-19;

* For mild cases

** Only for treatment in the first 48 hours of the clinical picture of flu-like illness or severe acute respiratory syndrome;

*** All cases that were considered moderate or severe with hospitalization;

**** Only for treatment in patients with pulmonary impairment;

***** Treatment in the initial phase of flu-like syndromes;

****** Prophylactic doses for critically ill hospitalized patients.

|  | Strong recommendation for |
| --- | --- |
|  | No absolute contraindication or “consider” or “can be offered” |
|  | “Under study”, “under assessment” or “unknown” |
|  | Conditional recommendation |
|  | Only in research settings |
|  | Conditional recommendation against |
|  | Compassionate use |
|  | Strong recommendation against |
|  | Only if secondary bacterial infection suspected |

^1^ Brasil. Ministério da Saúde. Secretaria de Atenção Primária à Saúde. Departamento de Ações Programáticas e Estratégicas. Manual de Recomendações para a Assistência à Gestante e Puérpera frente à Pandemia de Covid-19. Brasília : Ministério da Saúde, 2020. Available from: <https://docs.bvsalud.org/biblioref/2021/05/1179850/manual-instrutivo-para-a-assistencia-a-gestante-e-puerpera-fre_zVNq7Ij.pdf>

^2^ Brasil. Ministério da Saúde. Secretaria de Atenção Primária à Saúde. Departamento de Ações Programáticas e Estratégicas. Manual de recomendações para a assistência à gestante e puérpera frente à pandemia de Covid-19. 2 ed. Brasília : Ministério da Saúde, 2021. Available from: <https://bvsms.saude.gov.br/bvs/publicacoes/manual_assistencia_gestante_puerpera_covid-19_2ed.pdf>

^3^ Brasil. Ministério da Saúde. Nota informativa nº 9/2020-SE/GAB/SE/MS, de 20 de maio de 2020. Orientações para manuseio medicamentoso precoce de pacientes com diagnóstico da COVID-19. Available from: <https://docs.bvsalud.org/biblioref/2020/05/1096794/orientacoes-manuseio-medicamentoso-covid19.pdf>.

^4^ BRASIL. MINISTÉRIO DA SAÚDE. Orientações do Ministério da Saúde para Manuseio Medicamentoso Precoce de Pacientes com Diagnóstico da COVID-19. Brasília, DF: Ministério da Saúde, 2020. Available from: <https://docs.bvsalud.org/biblioref/2020/06/1102269/covid-final-16junho-livreto-1-v3.pdf>.

^5^ Brasil. Ministério da Saúde. Nota informativa nº 17/2020-SE/GAB/SE/MS, de 30 de julho de 2020. Orientações para manuseio medicamentoso precoce de pacientes com diagnóstico da COVID-19. Brasília: Ministério da Saúde, 2020. Available from: <https://bit.ly/3q7w5JT>.

^6^ FEBRASGO. Protocolo de Atendimento no Parto, Puerpério e Abortamento durante a Pandemia da COVID-19, 27 abr 2020. Available from: <https://www.febrasgo.org.br/pt/covid19/item/1028-protocolo-de-atendimento-no-parto-puerperio-e-abortamento-durante-a-pandemia-da-covid-19>.

^7^ FRABRASGO. Hidroxicloroquina e gestação. 01 jun 2020. Available from: <https://www.febrasgo.org.br/pt/covid19/item/1049-covid-19-hidroxicloroquina-e-gestacao>.

Supplementary Table 2: Risk factor estimates for hospitalization due to COVID-19 exclusively considering cases diagnosed by polymerase chain reaction (PCR). Multivariable analysis was performed after the selection of significant variables for the model.

| Risk factor | Univariate analysis  (N=220^a^) | | Full model multivariable analysis (N=220^a^) | | Reduced model multivariable analysis (N=220^a^) | |
| --- | --- | --- | --- | --- | --- | --- |
|  | OR | 95% CI | OR | 95% CI | OR | 95% CI |
| At least 1 dose of COVID-19 vaccine | 0.11 | 0.02 – 0.51 | 0.12 | 0.03 – 0.54 | 0.11 | 0.02 – 0.51 |
| Pre- or gestational hypertensive disorders | 2.77 | 0.89 – 8.63 | 1.97 | 0.58 – 6.69 |  |  |
| Maternal age >35 years | 2.59 | 1.02 – 6.56 | 1.93 | 0.70 – 5.38 |  |  |
| Pre- or gestational diabetes | 2.66 | 0.90 – 7.81 | 1.78 | 0.54 – 5.92 |  |  |
| Gestational age at infection >196 days | 1.64 | 0.65 – 2.54 |  |  |  |  |
| Preexisting pulmonary, cardiac, and renal conditions | 1.23 | 0.26 – 5.95 |  |  |  |  |
| Married/committed relationship | 0.90 | 0.32 – 2.54 |  |  |  |  |
| Nulliparity | 0.95 | 0.40 – 2.27 |  |  |  |  |
| University degree | 0.55 | 0.20 – 1.55 |  |  |  |  |
| Multiple pregnancy^b^ | – | – |  |  |  |  |

^a^ Estimates were computed under multiple imputations to missing data.

^b^ Not estimated due to low frequency
Full model includes all significant covariates from the univariate analyses (p-value<0.25). The reduced model includes all variables that remain after the selection process (p-value<0.1 or change in other estimates >20%).
The reference category is “No” for all risk factors, unless otherwise stated. For marital status, single never married or divorced was the reference group. For education, High School/ Primary school was the reference group.
